# Distribution of Attention Modulates Salience Signals in Early Visual Cortex

**DOI:** 10.1371/journal.pone.0020379

**Published:** 2011-05-26

**Authors:** Manon Mulckhuyse, Artem V. Belopolsky, Dirk Heslenfeld, Durk Talsma, Jan Theeuwes

**Affiliations:** 1 Vrije Universiteit, Amsterdam, The Netherlands; 2 Ghent University, Ghent, Belgium; University of Leuven, Belgium

## Abstract

Previous research has shown that the extent to which people spread attention across the visual field plays a crucial role in visual selection and the occurrence of bottom-up driven attentional capture. Consistent with previous findings, we show that when attention was diffusely distributed across the visual field while searching for a shape singleton, an irrelevant salient color singleton captured attention. However, while using the very same displays and task, no capture was observed when observers initially focused their attention at the center of the display. Using event-related fMRI, we examined the modulation of retinotopic activity related to attentional capture in early visual areas. Because the sensory display characteristics were identical in both conditions, we were able to isolate the brain activity associated with exogenous attentional capture. The results show that spreading of attention leads to increased bottom-up exogenous capture and increased activity in visual area V3 but not in V2 and V1.

## Introduction

One of the most debated issues in selective attention research is whether we are able to exert full attentional control over what we select from our environment. Attentional selection may either be controlled by the salience of objects present in the environment or by intentions, goals and beliefs of the observer. When, independent of the observer's goals and beliefs, specific properties present in the visual field determine the selection priority, selection is said to occur in an involuntary, bottom-up, stimulus-driven manner. When an observer intentionally and volitionally selects only those objects required for the task, selection is said to occur in a top-down, voluntary, goal-directed manner (see for a recent review [1]). Bottom-up and top-down control of attention represent the interplay of exogenous and endogenous neural activity patterns within the cortex. From a neurophysiological point of view, it can be assumed that stimulus-driven signals are combined with goal-driven signals at several cortical and subcortical levels [2]–[4].

Imagine a situation in which the visual system is confronted with two unique stimuli presented at different locations (i.e., singletons). One of the stimuli is task irrelevant but highly salient and one of the stimuli is less salient but highly task relevant. Within the system these two objects are in competition and the question is which object is going to win this competition for representation throughout the visual system. In line with the biased competition model of Desimone and Duncan [5] attention biases competitive interactions such that attended stimuli receive priority over unattended stimuli. Objects that are highly salient and stand out from the background may immediately receive attentional priority. Indeed, it is likely that before goal-driven signals can have an effect, the visual system is biased towards salient stimuli that resolve the competition simply on the basis of sensory input [1], [6]–[11]. This type of selection is basically exogenous and automatic and is often referred to as attentional capture.

Another way to bias the competition within the visual system is through goal-driven signals. Directing attention voluntarily to a location in space increases the sensory gain for features at that location and appears to alter the apparent stimulus contrast [12], [13]. These results imply that directing attention to a location results in a greater neuronal sensitivity [14]. This type of selection is endogenous and is accomplished through top-down signals that depend on the goal of the observer [15]–[20].

One way to conceive the interaction between exogenous and endogenous attentional control is to assume that the extent to which attention is voluntarily spread across the visual field affects the competition between objects. For example, Theeuwes [21] showed that abrupt onsets, which are known to capture attention exogenously, cease to capture attention when before display onset observers focus their attention to a limited area in space [22]. Consistent with biased competition, it is assumed that the consequences of directing spatial attention biases information processing in favor of stimuli appearing at the attended location at the expense of processing stimuli at unattended locations. Based on the notion that the focus of attention may play an important role in mitigating the effect of attentional capture, Theeuwes ([23], p.436) suggested that "top-down control over visual selection can be accomplished by endogenously varying the spatial attentional window” [24]–[26]. The notion is that the distribution of attention across the visual field, referred to as the attentional window, could be one of the factors explaining why salient color singletons sometimes fail to capture attention [27] while in other studies they do capture attention [9]. Belopolsky et al. [28] tested this idea directly. They adopted the original Jonides and Yantis [27] paradigm, in which participants had to serially search for a target letter, which could have a unique color at chance level. The size of the attentional window was manipulated by asking participants to detect either a local shape (focused attention) or a global shape (diffuse attention) before starting the search for a non-singleton target. The results showed that when attention was initially focused in the center there was no attentional capture while capture was observed when observers spread their attention across the visual field. Belopolsky et al. concluded that the attentional window can be varied in a top-down manner, either by anticipating serial search [27] or by instruction determined by the task set. Both manners will prevent attentional capture.

In a follow-up study, Belopolsky and Theeuwes [29] used the classic additional singleton task of Theeuwes [9] in which observers have to search for a shape singleton in which the target is presented while an additional salient distractor (a color singleton) is either present or absent. Typically, in this paradigm reaction times are longer when the salient distractor is present relative to when it is absent. This reaction time difference is explained in terms of attentional capture: before attention can move to the target, attention is exogenously -against the intentions of the observer- captured by the salient yet irrelevant color singleton [1], [9], [10], [25]. The innovation of Belopolsky and Theeuwes [29] was that while using this paradigm, they explicitly manipulated the extent to which attention was distributed across the visual field. In their experiment, observers only responded to the target when a specific go-signal was present. This go-signal could either be a particular letter in an RSVP stream presented at the center of the display, or it could be a particular global shape which was made up by the display elements. Therefore, before search started attention was either focused or distributed across the display. When attention was distributed across the visual field, the classic attentional capture effect was observed. That is, longer reaction times when a color singleton distractor was present relative to when it was absent. However, while using exactly the same visual displays capture was abolished when the size of the attentional window was reduced by instructing observers to direct their attention to the center of the display. In this case, reaction times did not differ between the color singleton present or absent conditions. This study demonstrates that the distribution of attention plays a crucial role in visual selection and in the occurrence of attentional capture [28], [30].

The present study took advantage of the fact that in Belopolsky and Theeuwes study [29] the visual displays in the diffuse and focused attention conditions were physically identical, with the former showing the classic capture effect and the latter not. Due to the fact that the displays were physically identical, this design allowed us to isolate the brain mechanisms associated with attentional capture. We isolated the response to the color singleton distractor by defining retinotopically corresponding regions of interest (ROIs) in early visual areas. Previous research has shown that top-down attention modulates sensory processing in early visual areas [15]–[20]. However, the modulation of sensory processing in early visual cortex due to bottom-up attention has not been investigated much, possibly due to difficulties in equating sensory input between conditions.

Notably, however, a few studies addressed the modulation of sensory processing due to stimulus-driven attentional capture. For example, Serences and colleagues [31], [32] examined capture of relevant distractors. They used peripheral color singletons that were presented during an RSVP task. The color singleton distractor could either match the target color and thus be part of the attentional set [33], [34] or the distractor could be of a different color. Behavioral results in both studies showed a decrease in performance only when the singleton distractor matched the target color. Consequently, the authors suggested that the decrease in performance reflected a shift of attention to the distractor. The fMRI results in Serences and Yantis [32] showed attentional modulation of distractor processing in parietal and frontal visual areas (IPS and FEF) but not in early visual areas. The fMRI results in Serences et al. [31] showed an attentional modulation of the relevant distractor in extrastriate cortex. Because only distractors that matched the target color modulated the response, it was suggested that attentional modulation in extrastriate cortex was due to reentrant feedback signals from IPS and FEF signaling the spatial location and features of visual stimuli that match the current attentional set. Note that in these studies capture by task-relevant distractors was examined. Therefore, the underlying neural processes might be different from studies examining task-irrelevant stimulus-driven capture.

Other studies examining bottom-up spatial attention used peripheral spatial cueing [35], [36] . In these studies, enhanced activity was observed at retinotopically corresponding target locations in striate and extrastriate cortex. Because the target locations were cued by a peripheral irrelevant onset cue, the enhanced activity was attributed to bottom-up attentional processing. Note, however, that these latter studies examined the effect of exogenous attention on target processing but could not isolate the brain activity associated with the exogenous capture of attention by a distractor itself. This was the purpose of the present study.

De Fockert et al. [37] investigated the modulation of distractor processing in a visual search task. In their fMRI study, they used a modification of the original additional singleton paradigm [9] in which the color singleton could either be the distractor or the target. This way, they contrasted the effects of color singleton distractor present versus absent with the effects of color singleton target present versus absent. A whole brain analyses showed that the presence versus absence of a color singleton target was not associated with enhanced brain activity, while the presence versus absence of a color singleton distractor was associated with enhanced activity in the parietal and frontal cortex. The authors suggested that the activity in the parietal cortex was associated with the involuntary shift of attention to the color singleton while the activity in the frontal cortex was associated with top-down control in order to resolve competition between color singleton distractor and target [38]. Although the study of De Fockert et al. [37] examined bottom-up driven attentional processes, it did not report enhanced activity in early visual areas. However, the design they used did not allow them to examine exogenously driven attentional sensory modulation of a salient stimulus without contrasting it with endogenously driven attentional modulation.

In the present study, the design made it possible to isolate the brain activity associated with exogenous attentional capture without contrasting it to endogenous spatial attention. Similar to Belopolsky and Theeuwes [29], we used a go-signal that instructed observers to either focus or distribute their attention before the onset of the search display. We expected that attentional capture would be associated with a greater response in early visual areas at the corresponding retinotopic locations of the color singleton distractor.

## Methods

### Participants

Thirteen healthy participants volunteered to take part in the fMRI experiment. All participants were right-handed and had normal or corrected-to-normal vision. Written informed consent was obtained before taking part in the experiment. Participants received a financial compensation. The protocol was approved by the ethical committee of the VU University Medical Center, Amsterdam, The Netherlands.

### Stimuli and Design

The behavioral task was basically the same as in Belopolsky and Theeuwes [29]. Stimulus presentation and response collection were controlled using E-Prime 1.1 (Psychology Software Tools). The stimuli were presented against a black background and consisted of a central RSVP stream and a search display presented around it. The RSVP stream consisted of 21 letters (sampled randomly from the pool of 18 letters, all letters of the alphabet except for G, M, O, Q, R, W and letters K and I that were used as a Go-signal). Each letter was gray and subtended 0.7°×0.8°, and was presented for 80 ms, followed by another 80 ms blank interval. The search display was positioned around the RSVP stream and consisted of 8 display elements that were equally spaced around it in a layout of either an imaginary circle (radius of 5.2°) or square (7.8°×7.8°). The search display was presented 2560 ms (16 letters) after the onset of the RSVP stream for 800 ms. Each search display contained a diamond element (3.1° in diagonal) presented among circles (2.3° in diameter). The diamond contained the target line-element (1.2°) that was oriented either horizontally or vertically. Participants had to respond to the orientation of this line-element by pressing a fiber-optic button with their right or left index finger. Each circle contained a line-element that was tilted 22.5° to either side of horizontal or vertical plane. The orientations of the line-elements inside the circles were chosen randomly. All search elements were green, except for the trials in the color singleton present condition on which one of the circles had a red color (approximately equiluminant). The difference in reaction time (RT) between the color singleton present and color singleton absent trials was used to measure attentional capture behaviorally.

**Figure 1:**
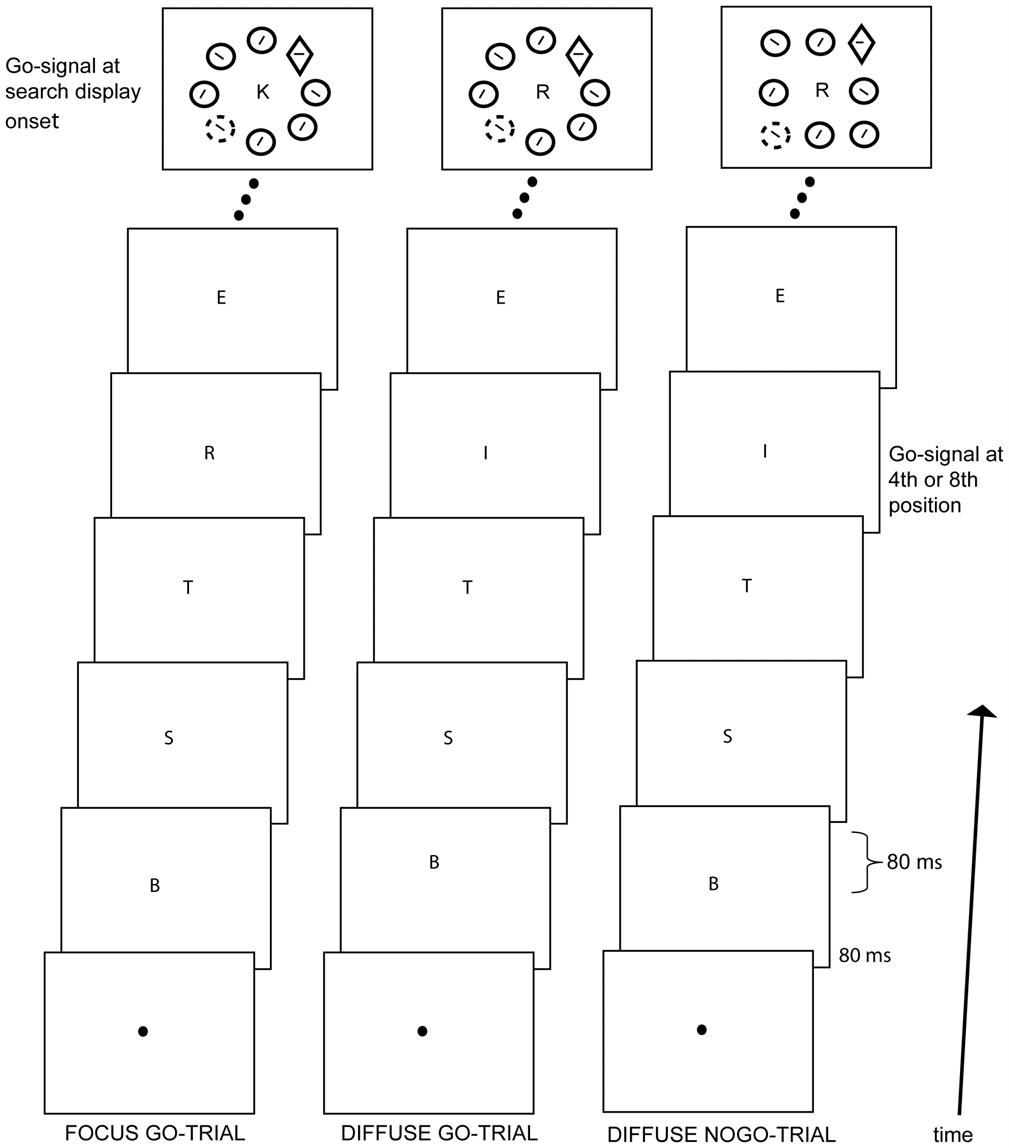
Layout of the currently used paradigm. From bottom to top, the succession of events within a trial is shown. Participants initially attended to the RSVP stream in the middle of the display. Depending on the condition, they either had to detect the letter “K” or the letter “I” that served as a go-signal. Left panel: an example stimulus sequence of the focused condition when a color singleton distractor (dotted circle) was present. After the letter K was presented (70% of all trials), participants had to respond to the line-element in the diamond. Note that the go-signal in the focused condition was presented at search display onset. Center panel: Shown here is an example of the diffuse attention condition when a color singleton distractor was present with the first go-signal I. After the letter I was presented (85% of all trials), participants had to respond to the line-element in the diamond but only when the display elements made up a circle. Right panel: When the display elements made up a square (15% of all trials), participants had to withhold their response. Note that the first go-signal in the diffuse condition was presented before search display onset.

The experiment consisted of two conditions that were run in separate blocks: the focused attention condition and the diffuse attention condition. The focused attention condition is illustrated in the left panel of Figure 1. In the focused attention condition, participants had to attend to the RSVP stream until they perceived the go-signal, the letter “K”. This letter was presented simultaneously with the onset of the search display, 2560 ms after RSVP onset (17^th^ position). The letter “K” signaled that participants had to respond to the line-element in the diamond. This way, participants were focused in the middle of the display when the search display was presented. Catch trials consisted of trials without this go-signal (30%) and although the search display was presented participants had to withhold their response.

The diffuse attention condition is illustrated in the center and right panels of Figure 1. In the diffuse attention condition, participants had to attend to the RSVP stream until they perceived the first go-signal, the letter “I”. This letter was presented either 480 ms (4^th^ position) or 1120 ms (8^th^ position) after RSVP onset. The letter “I” signaled that attention had to be directed to the global shape of the upcoming search display and the RSVP stream in the middle could be ignored. If the global shape of the elements in the search display made up a circle (center panel of Figure 1), participants searched for the diamond shape and responded to the orientation of the line segment inside the diamond shape. However, if the global shape of the elements in the search display made up a square (right column Figure 1), participants had to withhold their response. In the diffuse attention condition there were two types of catch trials: Catch trials in which the go-signal letter was not presented (15% of trials) and catch trials in which the go-signal letter was presented but the global shape was a square (15% of trials). Note that the length of the RSVP stream in the diffuse condition equals the length of the RSVP stream in the focused condition. In addition, the letter “I” was never presented in the focused condition and that the letter “K” was never presented in the diffuse condition to avoid confusion.

### Procedure

Participants practiced both conditions outside the scanner until they had more than 80% correct responses. Participants were explicitly told to keep their eyes fixated at the center of the display. In the scanner, the participants' head was immobilized using foam pads to reduce motion artifact and earplugs were used to moderate scanner noise. Half of the participants started with the diffuse attention condition, the other half with the focused attention condition. Each condition was presented in four successive blocks. Of the four blocks, one block consisted of color singleton absent trials. Half of the subjects started with a color singleton absent block, the other half ended with a color singleton absent block. The color singleton absent trials were only used for the analysis of behavioral data. Each block consisted of 36 response trials and 18 catch trials. The color singleton could be presented at four different locations, which were counterbalanced within a block. The target was randomly presented at one of the other locations but never next to the distractor location. Besides the catch and the regular trials, each block contained 27 no-stim trials of the same duration as the regular trials. These trials were included to avoid saturation of the hemodynamic blood-oxygen level dependent (BOLD) signal and to vary the onset of each trial according to a random exponential distribution that allowed us to estimate the BOLD response to each event of interest [39]. All trial types were presented in a randomized first-order counterbalanced sequence, in which each trial type was preceded equally often by every trial type in the design. After participants performed the experimental task, they received two retinotopic mapping tasks and a 3-D anatomical scan.

### Scan acquisition

Imaging sessions took place in a 1.5 T Siemens Sonata scanner (Siemens Medical Systems, Erlangen, Germany), using an 8-channel phased-array head coil. Visual stimuli were back-projected onto a screen that was viewed by the participants through an angled mirror positioned on top of the head coil.

Functional data were collected using an EPI sequence scanning the whole brain in 25 near-axial slices. Scanning parameters for the main task were: TR = 2560 ms, TE = 60 ms, flip angle  = 90°, slice thickness  = 4 mm, gap = 0.8 mm, acquisition matrix  = 64×64, and in-plane resolution  = 3.1×3.1 mm. All volumes were on-line motion corrected.

Scanning parameters for the retinotopic mapping tasks were: TR = 2280 ms, TE =  ms, flip angle  = 90°, slice thickness  = 3 mm, gap = 0.6 mm, acquisition matrix  = 64×64, and in-plane resolution  = 3.1×3.11 mm. All volumes were on-line motion corrected.

A 3-D anatomical scan was made at the end of the session, using a T1-weighted MP-Rage sequence. Scanning parameters were: TR = 2730 ms, TE = 3.43, TI = 1000 ms, flip angle  = 7°, sagittal slice thickness  = 1 mm, acquisition matrix  = 256×224 pixels, in-plane resolution  = 1 mm×1 mm.

Electro- oculogram (EOG) was recorded in the scanner between 2 carbon electrodes placed at the outer canthi of each eye to monitor eye movements during the MRI sessions.

### Retinotopic mapping

In two additional blocks, a polar mapping was performed to identify the borders between visual areas V1, V2, V3 by stimulating the four distractor locations with local flickering checkerboard patterns. The checkerboard patterns were counterphased at 10 Hz, each stimulus lasted 4 s and was followed by the next after 8 s. These localizer blocks served to identify the exact projection of the distractor locations within each visual area.

### MRI data analysis

**Figure 2:**
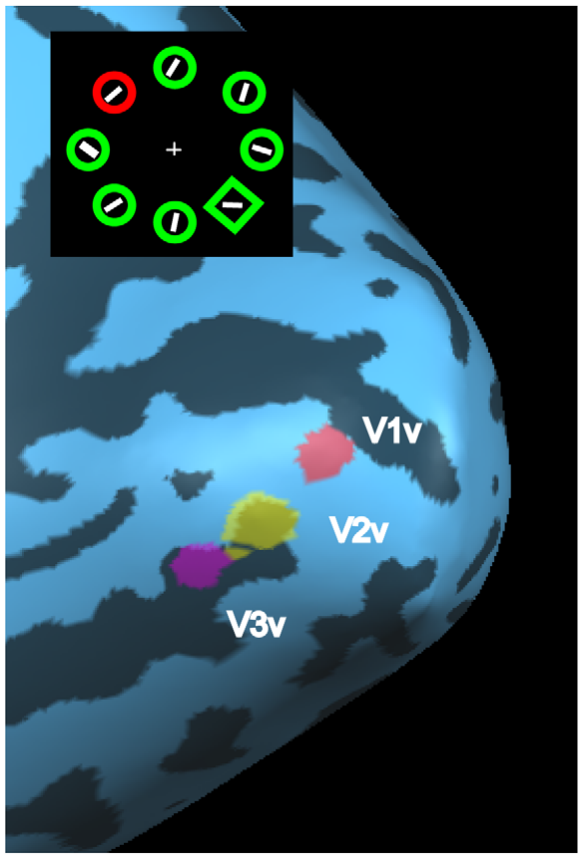
ROIs in early visual areas V1, V2 and V3. Shown are the ROIs in the right hemisphere of one participant for the distractor location in the upper left visual field, as determined by our mapping procedure.

MRI data were analyzed using BrainVoyager QX 2.1 (Brain Innovation, Maastricht, The Netherlands). The first two volumes of each block were omitted in order to avoid differences in T1 saturation. The preprocessing of the remaining functional volumes consisted of slice scan-time correction, highpass filtering (0.01 Hz), slight spatial smoothing (3 mm FWHM Gaussian kernel), but no temporal smoothing. The functional scans were automatically or manually coregistered to each individual anatomical scan and converted to Talairach space [40]. Anatomical scans were also converted to Talairach space and segmented. A model of the cortical surface was created based on the boundary between gray and white matter. The segmented brains were then inflated onto which the functional retinotopical data were projected. Based on the polar mapping, the borders of early visual areas (V1d, V2d, V3d and V1v, V2v, V3v) in each hemisphere were defined. On the basis of the data obtained from the retinotopic mapping, the activated regions within the early visual areas were defined as ROIs (see Figure 2). In one of the participants it was not possible to localize the ROIs. Therefore, this participant was excluded from any further analyses.

The functional MRI time series were averaged within each ROI and then further analyzed. Color singleton absent trials and catch trials were not analyzed because there were too few trials. Data was collapsed across the four locations for each visual area to increase signal to noise ratio. The averages were baseline corrected from -1 TR (-2560 ms) to search display onset. The average of the peak and TR 1, 2 and 3, were used to perform statistical analysis.

## Results

### EOG data

For each run and each participant we calculated the variance of the recorded EOG signal. If there were no systematic eye movements, the variance of the EOG should be the same for each block. Participants were excluded from further analyses if they showed a standard deviation in one or more runs that was larger than 1.5 times the overall mean standard deviation. This resulted in the exclusion of two participants. Of the remaining 10 participants, a two-related Wilcoxon test showed that there were no systematic differences in eye movements between the diffuse attention and the focused attention condition (z = .478, N-ties = 0, *p*>0.6).

### Behavioral results

Reaction times below or above two standard deviations from the group mean in each condition and incorrect responses were omitted from analyses. The error data in the experimental trials between the focused color singleton absent (9.6%), focused color singleton present (9.6%), diffuse color singleton absent (7.8%) and diffuse color singleton present (8.2%) did not differ significantly from each other (*p* = .74). In addition, the error data in the catch trials for the focused condition (3.8%) and the diffuse No “I” presentation (4.7%) and diffuse square display presentation (6.1%) conditions did not differ significantly (*p* = .8).

**Figure 3:**
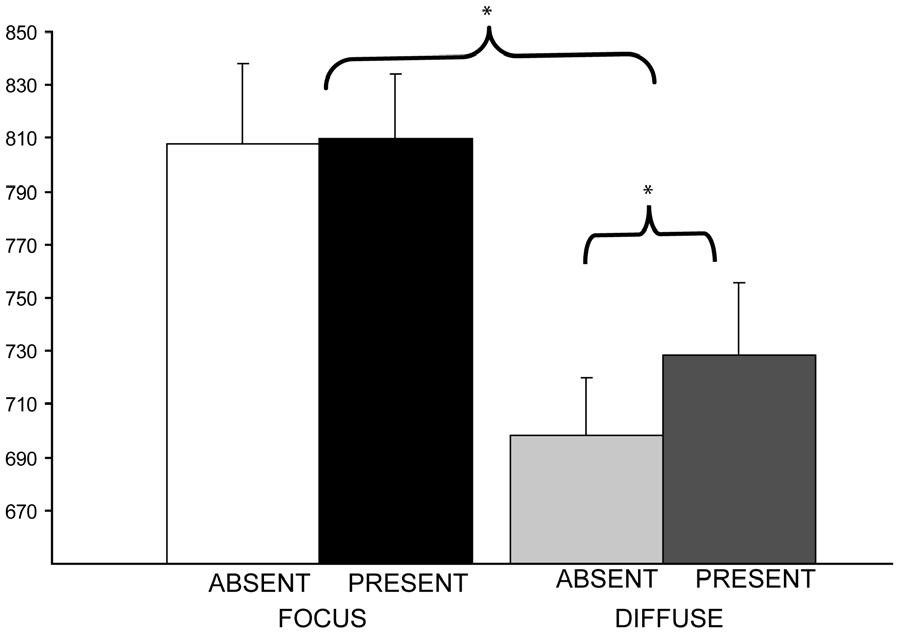
Behavioral performance during scanning. Bars represent mean reaction times with color singleton present (dark bars) and absent (light bars) for the diffuse attention and the focused attention condition. Asterisks indicate significant differences between the focused and the diffuse condition and between the absent and present trials in the diffuse condition.

To investigate the effect of the color singleton distractor in the focused and diffuse conditions, we performed a 2×2 repeated measures ANOVA with attentional window (focused and diffuse attention) and color singleton distractor (absent and present) as factors. As can be seen in Figure 3, the results revealed a main effect of attentional window (F(1, 9)  = 55.18, *p*<0.01) due to significantly faster reaction times in the diffuse attention condition compared to the focused attention condition. There was neither a main effect of color singleton distractor (*p* = .19) nor an interaction between attentional window and color singleton distractor (*p* = .17). Because the absence of a significant interaction could have been due to insufficient statistical power, we conducted planned comparisons between the color singleton present and absent trials for the diffuse and for the focused attention condition. These planned comparisons showed that in the focused attention condition mean reaction time did not differ between the color singleton distractor absent (808 ms, SE 30 ms) and present (810 ms, SE 24 ms; t<1), whereas in the diffuse attention condition, color singleton distractor absent (698 ms, SE 22 ms) and present (729 ms, SE 27 ms) did differ (*t* (9)  = 3.41, *p*<0.01). This is consistent with the result obtained in the Belopolsky & Theeuwes [29] study.

### fMRI results

**Figure 4:**
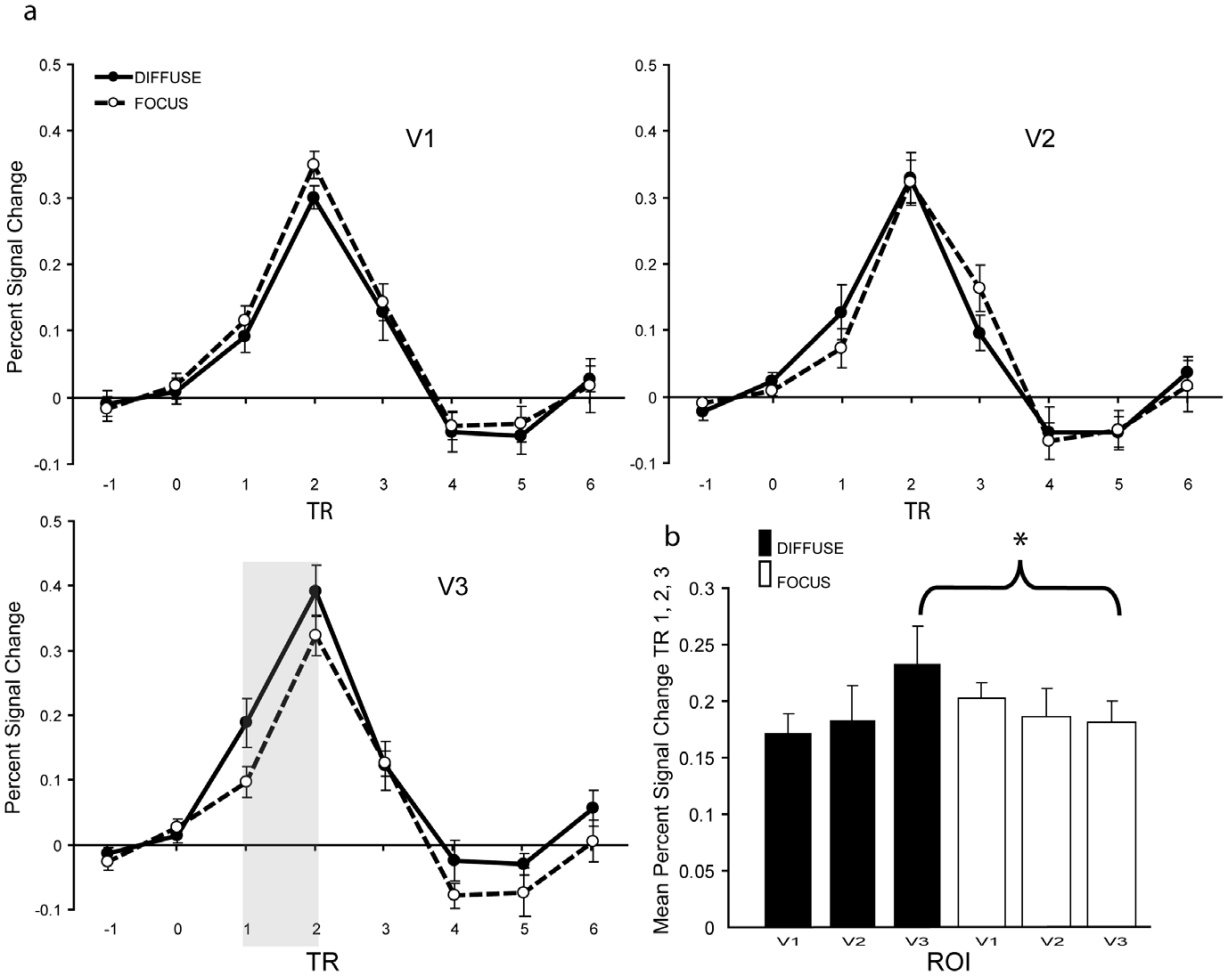
(a) Event related averages of the fMRI responses. Shown are the averages to the color singleton in the diffuse attention (solid line) and the focused attention condition (dotted line) in V1, V2 and V3, collapsed across quadrants and participants. In V3, activity in response to the color singleton was significantly higher at TR1 and TR2 (shaded area) in the diffuse attention condition relative to the focused attention condition. (b) Mean percent signal change in V1, V2 and V3. Shown are mean signal change collapsed over TR1, TR2 and TR3 for the diffuse attention (black bars) and focused attention condition (white bars). In V3, mean activity in the diffuse condition in response to the color singleton was significantly higher than mean activity in the focused condition. In addition, the activity induced by the color singleton increased from V1 to V3 when the attentional window was wide.

Figure 4a shows the event-related averages of the fMRI responses to the color singleton distractor in the focused and diffuse attention condition for each visual area, collapsed across quadrants and participants. A 3×3×2 repeated measured ANOVA with TR (1, 2 and 3), ROI (V1, V2 and V3) and attentional window (focused and diffuse attention condition) as factors showed no main effects of attentional window or ROI (both F<1), a main effect of TR (F(2, 18)  = 40.94, *p*<0.01, Huynh-Feldt corrected), a marginal significant three-way interaction (F(4, 36)  = 2.76, *p* = 0.06, Huynh-Feldt corrected), no interaction between TR and ROI ( *p* = 0.34), an interaction between TR and attentional window (F(2, 18)  = 4.92, *p*<0.05, Huynh-Feldt corrected) and an interaction between ROI and attentional window (F(2, 18)  = 4.29, *p*<0.05, Huynh-Feldt corrected). To further investigate the responses for each ROI separately, we performed ANOVAs with TR (1, 2, 3) and attentional window (focused and diffuse attention condition) as factors. There was neither an interaction nor a main effect of attentional window in V1 (both *p*>0.1). In V2 we found no main effect of attentional window (F<1) but an interaction between attentional window and TR (F(2, 18)  = 6.06, *p*<0.05, Huynh-Feldt corrected). Post-hoc comparisons showed that at the first TR, activity was larger in the diffuse attention condition compared to the focused attention condition (*t* (9)  = 2.25, *p = *0.05). In V3, we found a marginally significant main effect of attentional window (F(1, 9)  = 3.68, *p* = 0.09) and an interaction between attentional window and TR (F(2, 18)  = 4.02, *p*<0.05, Huynh-Feldt corrected). Post-hoc comparisons showed that activity was significantly larger in the diffuse attention condition compared to the focused condition at the first TR (*t* (9)  = 2.71, *p<*0.05) and at the second TR (*t* (9)  = 2.1, *p<*0.05, one-tailed). These results show a modulation of the BOLD response by the color singleton distractor depending on whether the attentional window was wide or narrow.

Because we did not make any predictions regarding temporal differences between the conditions, we additionally performed the same analyses for the average of the BOLD response peak (TR 1, TR2 and TR3). An ANOVA with ROI (V1, V2 and V3) and attentional window (focused and diffuse attention condition) as factors showed no main effect of ROI or attentional window (both F<1), but an interaction between ROI and attentional window (F(2, 18)  = 4.29, *p*<0.05, Huynh-Feldt corrected). Post-hoc comparisons showed that this interaction was due to a significant larger activity in the diffuse attention condition compared to the focused attention condition only in V3 (*t* (9)  = 1.9, *p<*0.05, one-tailed). To further investigate the activity related to the color singleton, we also tested for a linear trend. Tests of the first and second order trends indicated that there were no main effects of ROI or attentional window (F<1), but there was an interaction between ROI and attentional window for the linear trend (F(1, 9)  = 7.76, *p*<0.05). Post-hoc tests showed that in the diffuse attention condition the activity tended to increase linearly across ROIs (F(1, 9)  = 4.07, *p* = 0.08) while it did not in the focused attention condition (F<1). The nonlinear trends were not significant (both F<1). These results suggest that activity induced by the color singleton increased linearly from V1 to V3 when the attentional window was wide.

## Discussion

This study shows that a salient irrelevant color singleton modulates visual processing in early visual areas. When the attentional window was wide, activity at the location of the irrelevant color singleton was enhanced compared to when the attentional window was narrow. Since we observed attentional capture in the diffuse attention condition and no attentional capture in the focused condition we attribute the enhanced retinotopic activity in V3 to bottom-up driven spatial attentional processes. Consistent with previous studies (for an overview see [1]) we assume that in the diffuse condition, attention is first shifted to the distractor before it is redirected to the target. In this case, the competition between the highly salient distractor and the less salient target was initially biased towards the distractor because of its higher bottom-up activation.

One could argue that not the attentional window was manipulated but rather task load. However, task load is very similar in both conditions. In the focused and diffuse task participants first have to identify a go-signal and then perform a search task. In both cases the task is most likely performed serially: in the focused attention condition as soon as the target letter is detected, attention can go to the search display and the RSVP no longer has to be attended; in the diffuse attention condition as soon as the global shape is identified the display can be searched. In addition, just as in Belopolsky & Theeuwes [29] attention conditions were blocked and participants knew before each trial whether they should diffuse or focus their attention. Therefore, just as in Belopolsky & Theeuwes [29], by the time the search display arrives participants' attention was either diffuse or focused. This led to differences in attentional capture and in the pattern of activation in visual cortex.

The enhanced retinotopic activity in V3 implies that exogenous spatial attention modulates sensory processing in a similar way as endogenous attention does. That is, several studies have demonstrated enhanced activity in early visual areas when attention is voluntarily directed to a location in space [15]–[19]. This increase is believed to reflect enhanced processing of attended stimuli as a result of sensory gain control [41], [42]. It is possible that sensory gain control is also the mechanism by which exogenous attention enhances processing of the attended stimulus. However, endogenous attentional effects are attributed to top-down signals from higher level areas to lower level areas [2], [17], [42], [43]. For example, it has been shown that top-down attention can modulate responses in early visual areas even in the absence of visual stimulation [19], [43], [44].

In our study, it is more likely that the attentional effects are the result of feedforward processing. Although the BOLD response does not have the temporal resolution to dissociate between early and late processes, single-cell recordings suggest that stimulus-driven attentional capture occurs during the initial feedforward sweep of information processing [45]. In an additional singleton task, similar to the present one, involving non-human primates, Ogawa and Komatsu showed a modulation in extrastriate cortex within the first 175 ms after stimulus onset that was not modulated by goal-driven attentional processes.

In addition, the idea that bottom-up driven attentional effects in visual cortex are the result of feedforward processing would be consistent with our finding that V1 was not modulated. The absence of an attentional effect in V1 is supported by a cueing study of Liu et al. [35]. They argued that the absence of an effect in V1 can be attributed to a lack of feedback signals from extrastriate cortex to striate cortex. Liu et al. reasoned that bottom-up attentional effects might be driven by a feedforward mechanism with an increase of attentional effects over visual areas. This idea is consistent with our results and suggests that while striate cortex codes for basic feature elements, extrastriate cortex is the first to reflect bottom-up attentional activity. A similar suggestion was done by Kincade et al. [46]. In an fMRI study investigating the differences between exogenous and endogenous cueing effects they found enhanced activity in extrastriate cortex induced by an exogenous cue compared to an endogenous cue. They suggested that this enhanced activity reflects the marking of a location of interest that is subsequently signaled to higher visual areas such as the frontal eye fields (FEF). FEF and higher visual areas such as parietal cortex are believed to serve as a salience map involved in bottom-up spatial selection [47], [48]. However, because of the slow BOLD response we cannot rule out the possibility that extrastriate cortex was modulated by other areas. For example, extrastriate cortex could be modulated by feedforward signals from subcortical areas such as the superior colliculus (SC) [49] or by signals from higher visual areas such as the FEF and parietal cortex, perhaps in the same way as in endogenous attention [50].

Although we attribute the enhanced retinotopic activity in V3 to exogenous spatial attention, it is possible that the neural response is partly modulated by a goal-driven attentional effect. That is, attention has to be redirected to the target after the irrelevant distractor has captured attention. In this scenario, the enhanced activity would reflect disengagement of attention [51]. However, while re-directing attention implies an active process that is mostly associated with enhanced activity in parietal cortex [2], [52] , one would expect this process to reduce sensory processing in visual cortex in order to re-orient to the target and not enhance sensory processing as we found in our study. Nevertheless, our paradigm does not distinguish processes related to attentional capture and processes related to disengagement of attention. Therefore, we cannot rule out the possibility that the enhanced activity in V3 partly reflects redirecting of spatial attention.

To our knowledge, this study is the first fMRI study that manipulated the attentional window as a method to investigate attentional capture. However, it is of interest that Kincade et al. [46] also found that a peripheral exogenous cue (one colored box among a display of several boxes) as well as a peripheral neutral cue (a display with several colored boxes) induced a greater response in occipital areas than an endogenous cue that was presented at fixation while the same display with colored boxes as in the neutral cue condition was presented. The authors proposed that this difference was due to the distribution of attention. In their study, attention was probably more distributed across the visual field in the exogenous and in the neutral condition than in the endogenous condition where the cue was presented at fixation. Consequently, due to the wider attentional window, the peripheral colored boxes that served either as an exogenous or neutral cue resulted in a stronger response than the same display in which the cue was presented at fixation.

Our results further show a linear increase over the ROIs when the attentional window was wide while activity stayed the same over the different ROIs when the attentional window was narrow. This is consistent with results from the spatial cueing study by Liu et al. [35]. Liu et al. found increased attentional effects along the hierarchy of visual areas. Moreover, the observed increase in activity in the diffuse attention condition and the absence of a change in activity in the focused condition implies that there is no evidence of active inhibition in this latter condition. That is, previous research showed that visual processing of distractors in the periphery is modulated by task load of the central task [53], [54]. This modulation is characterized by a decrease of activity over visual areas as a consequence of task difficulty [54]. Therefore, the absence of a change in activity in the focused condition and a linear increase in activity in the diffuse condition together argue against the idea that differences between the two conditions can be attributed to a difference in task difficulty or to inhibitory processes of the distractor location in the focused condition.

Although our results give more insight into the neural processes underlying bottom-up attentional capture, the neural processes underlying resistance to attentional capture are not yet clear. Resistance to attentional capture has been previously associated with activity in frontal cortex [37], [38]. De Fockert et al. suggested that top-down control by the frontal cortex either reflects maintenance of priorities between relevant and irrelevant stimuli or active inhibition of distracting stimuli. An fMRI study by Talsma et al. [38] showed that an increase in frontal activity was associated with a smaller attentional capture effect. Our results suggest that active inhibition of the distractor location is not essential to prevent attentional capture. In a recent study, Leber [55] also suggested that the frontal cortex plays a role in resistance to attentional capture. In this fMRI study, observers were presented with the additional singleton paradigm while pre-trial activity was measured. The results showed that pre-trial activity in middle frontal gyrus (MFG) could predict whether or not the upcoming salient color singleton would capture attention. A greater response in MFG was correlated with stronger top-down control resulting in a smaller capture effect. In addition, by varying set-size it was ruled out that resistance to attentional capture resulted from a slower serial search strategy because set-size had no influence on the attentional capture effect. However, how MFG asserts top-down control over visual selection is not yet clear.

In conclusion: The present study shows that stimulus-driven attentional capture is associated with increased retinotopic activity in extrastriate visual cortex. Moreover, this enhanced activity increased linearly along the hierarchy of visual areas and reached significance in visual area V3.
